# Novel Lipid Formulation Increases Absorption of Oral Cannabidiol (CBD)

**DOI:** 10.3390/pharmaceutics16121537

**Published:** 2024-12-01

**Authors:** Edward Chesney, Ndabezinhle Mazibuko, Dominic Oliver, Amedeo Minichino, Ayşe Doğa Lamper, Lucy Chester, Thomas J. Reilly, Millie Lloyd, Matilda Kråkström, Alex M. Dickens, Matej Orešič, Eric Lynch, Gregory Stoloff, Mitul A. Mehta, Philip McGuire

**Affiliations:** 1 Department of Psychosis Studies, Institute of Psychiatry, Psychology and Neuroscience, King’s College London, London SE5 8AB, UK; 2 National Addiction Centre, Institute of Psychiatry, Psychology and Neuroscience, King’s College London, London SE5 8AF, UK; 3 South London and Maudsley NHS Foundation Trust, London SE5 8AZ, UK; 4 Department of Neuroimaging, Institute of Psychiatry, Psychology and Neuroscience, King’s College London, London SE5 8AF, UK; 5 The Centre for Innovative Therapeutics (C-FIT), Institute of Psychiatry, Psychology and Neuroscience, King’s College London, London SE5 8AF, UK; 6 Department of Psychiatry, Oxford University, Warneford Hospital, Oxford OX3 7JX, UK; 7 NIHR Oxford Health Biomedical Research Centre, Oxford OX3 7JX, UK; 8 Research Centre, Centre Hospitalier de l’Université de Montréal (CRCHUM), Montréal, QC H2X 0A9, Canada; 9 Department of Psychiatry and Addiction, Faculty of Medicine, Université de Montréal, Montréal, QC H2X 0A9, Canada; 10 Turku Bioscience Center, University of Turku and Åbo Akademi University, 20520 Turku, Finland; 11 Department of Chemistry, University of Turku, 20014 Turku, Finland; 12 School of Medical Sciences, Örebro University, 701 82 Örebro, Sweden; 13 SEEK Group, London EC4N 8AF, UK

**Keywords:** CBD, cannabidiol, pharmacokinetics, bioavailability, formulation

## Abstract

**Background**: Cannabidiol (CBD) is an approved treatment for childhood epilepsies and a candidate treatment for several other CNS disorders. However, it has poor oral bioavailability. We investigated the effect of a novel lipid formulation on its absorption in humans and on its tissue distribution in mice. **Methods**: In a double-blind crossover study in fasting healthy volunteers, we compared the pharmacokinetics of a single dose of 1000 mg of CBD in the lipid formulation and in a powder formulation (ClinicalTrials.gov: NCT05032807). In a second study, male CD1 mice were administered CBD in either the lipid formulation or dissolved in water, via oral gavage (n = 1 per timepoint). The tissue distribution of CBD was assessed using matrix-assisted laser desorption/ionization mass spectrometric imaging. **Results**: Plasma exposure (AUC_0–48_) of CBD was nine times greater for the lipid formulation than the powder formulation (611.1 ng·h/mL [coefficient of variation {CV%}: 104.6] and 66.8 ng·h/mL [CV%: 50.7], respectively). With the powder formulation, the AUC_0–48_ was related to the concentration of specific gastrointestinal bacteria and bile acids. These associations were attenuated with the lipid formulation. In the animal study, after treatment with the lipid formulation, measurable concentrations of CBD were identified in all organs. For the aqueous formulation, tissue concentrations of CBD were below the limit of quantification. **Conclusions**: Administering oral CBD in a lipid formulation was associated with an increase in its gastrointestinal absorption, as well as an attenuation of the relationship between its absorption and features of the gut microbiome.

## 1. Introduction

Cannabidiol (CBD) is an approved treatment for childhood epilepsies [1] and a promising candidate treatment for psychosis, Parkinson’s disease, anxiety, addiction, and insomnia [2,3,4,5,6,7,8,9]. It has a relatively benign adverse effect profile and is highly accepted by patients compared to existing psychiatric treatments [10,11]. CBD is a highly lipophilic molecule that is not readily absorbed when administered orally, and undergoes substantial first-pass hepatic metabolism. As a result, it has poor oral bioavailability (5–10%) [12]. However, this can be improved if CBD is administered with food, especially food with a high fat content [13,14,15].

The only form of CBD approved for clinical use is Epidiolex, which is formulated with sesame oil. The latter is mainly composed of polyunsaturated fatty acids such as linoleic and oleic acid, and increases CBD’s bioavailability [12,13]. Izgelov and colleagues compared the effects of CBD dissolved in sesame oil and CBD in a ‘self-nano-emulsifying drug delivery system’ (SNEDDS) on its bioavailability. Relative to a powder formulation of CBD, the sesame oil formulation increased its peak concentration (C_max_) by 17× and the plasma exposure over time (AUC_t_) by 8×, while the SNEDDS formulation increased the C_max_ by 22× and the AUC by 7× [16]. Knaub and colleagues found that, compared to a medium-chain triglyceride formulation, a ‘novel self-emulsifying drug delivery system’ increased the C_max_ by 4.4× and the AUC by 1.8–2.9× [17].

The pharmacokinetic properties of CBD may also affect its distribution and ability to target organs. A few studies have investigated the tissue distribution of CBD after a single dose [18,19,20,21]. Child and Tallon administered 0, 30, 115, or 230 mg/kg CBD (dissolved in medium chain triglyceride oil) to adult male and female rats (n = 6/group) once daily for 28 days [22]. The concentrations of CBD in adipose tissues were 10–100× higher than in either liver or skeletal muscle.

Here, we investigated whether a lipid formulation that has previously been used to enhance the oral absorption of ibuprofen [23] could enhance the bioavailability of CBD. The formulation, which is solid at room temperature, contains Maisine^®^ CC, Gelucire^®^ 43/01, and polyethylene glycol 400, which have been melted and mixed with CBD. All ingredients used in the formulation have been used as inactive ingredients in previous approved medicines in the same or lower concentrations. We first examined its effects on healthy volunteers, and tested the hypothesis that the absorption of CBD, as indexed by its C_max_ and the AUC, would be significantly increased by administration in the lipid formulation.

The gut microbiome is understood to have an important role in drug absorption and metabolism [24]. Bacteria can produce secondary bile acids, which aid in the formation of micelles and may therefore influence the absorption of lipophilic drugs such as CBD [24]. Commensal gut bacteria can also affect the activity of CYP3A4, an enzyme involved in CBD metabolism [25]. To investigate the contribution of the gut microbiome to inter-individual variations in the absorption of CBD [26,27], we collected stool samples before the administration of the lipid and the control formulations. Because some effects of CBD may be mediated by endocannabinoids [28,29], we measured the plasma concentrations of endocannabinoids, as well as those of CBD and its metabolites.

We then conducted a parallel study in mice to investigate the effect of the lipid formulation on the distribution of CBD to putative target organs. Matrix-assisted laser desorption/ionization (MALDI) mass spectrometric imaging was used to assess the tissue distribution of CBD at steady state [30], comparing the plasma and whole-body pharmacokinetics of CBD after repeat dosing. We compared the lipid formulation with a formulation in which CBD was dissolved in water. Our hypotheses were that administering CBD in the lipid formulation would be associated with increased concentrations of CBD in the brain and peripheral tissues, as well as with increased gastrointestinal absorption (as indexed by its C_max_ and the AUC).

## 2. Materials and Methods—Study 1

### 2.1. Design

This was a phase 1, double-blind, two-period crossover study in healthy volunteers in the fasting state, comparing a novel formulation of CBD in a lipid matrix and a standard CBD-only powder formulation. The procedures included a screening visit followed by two experimental visits, each lasting 48 h and separated by a minimum 2-week washout period, and a follow-up assessment after 7–14 days. All trial procedures were conducted from June to August 2022 at the National Institute for Health Research King’s Clinical Research Facility at King’s College Hospital, London, UK.

### 2.2. Inclusion and Exclusion Criteria

The study recruited healthy volunteers aged between 18 and 45 years. Healthy volunteer status was determined through clinical history, physical examination, electrocardiogram (ECG), vital signs, and laboratory tests of blood and urine. Key exclusion criteria included prescribed medication (apart from contraceptives), the use of any products containing CBD within the past six months, the use of any over-the-counter medications or health supplements within the past 2 weeks, the intake of more than 14 units of alcohol weekly or 10 cigarettes per day, the use of any illicit substances within the last six months, BMI < 18 or >30.0 kg/m^2^, pregnancy or breastfeeding, and not being willing to use adequate contraception.

### 2.3. Screening Visit

After written informed consent was obtained, the participants provided their demographic information and medical history. The investigations included the following: physical examination; measurement of height, weight and body fat; an alcohol breath test; a urine sample for urinalysis, pregnancy and illicit drug testing; phlebotomy for liver function tests (LFTs), urea and electrolytes (U&Es) and full blood count (FBC); and an ECG.

### 2.4. Randomisation and Blinding

The randomisation system was provided by King’s Clinical Trials Unit. A block randomisation with a block size of two was used. Both participants and researchers were blinded to treatment allocation and the two formulations had an identical appearance. Unblinding was completed after database lock.

### 2.5. Fasting Requirements

Participants were only allowed to drink water from 10 p.m. the evening prior to drug administration until 1 pm the following day. To confirm the participants’ fasting status, their serum bile acids were measured locally at Synnovis, King’s College Hospital (London, UK), using a Roche Cobas analyser.

### 2.6. Study Drug

Both formulations contained naturally derived CBD processed into a highly pure crystalline powder by BSPG Laboratories (Sandwich, Kent, UK). Analysis of the CBD demonstrated that impurities were negligible, and that no delta-9-tetrahydrocannabinol was detectable. The lipid CBD formulation contained Maisine^®^ CC, a winterised oil composed of long-chain mono-, di-, and triglycerides; Gelucire^®^ 43/01, a glyceride used as a matrix agent and a viscosity-increasing agent; and polyethylene glycol 400. The comparator comprised CBD powder only. Identical hydroxypropyl methylcellulose capsules were used for both formulations, with a dose of 1000 mg administered using 5 capsules containing 200 mg of CBD each [1,9].

### 2.7. Experimental Procedure

Participants’ eligibility for the study was reviewed at the start of each experimental visit. A urine drug screen and alcohol breath test were performed and adherence to the fasting protocol was confirmed through a self-report and the testing of serum bile acids [31].

### 2.8. Blood and Stool Sampling, Processing and Analysis

Blood samples were collected at the following time-points via an intravenous cannula into 5 mL EDTA tubes: pre-dose, 0.5, 1, 2, 3, 4, 5, 6, 8, 24, and 48 h post-dose. Samples were centrifuged within 10 min of collection (3000 rpm for 10 min at 4 °C). After centrifugation, the plasma was decanted into cryovials and immediately placed in a −20 °C freezer before being transferred to a −80 °C freezer at the end of each day.

At the end of the study, plasma samples were transported on dry ice via a temperature-controlled courier to the Turku Metabolomics Centre (Turku Bioscience Centre, Finland). Plasma samples were analysed for CBD, 6-OH-CBD, 7-OH-CBD, 7-COOH-CBD, THC and THC-COOH, as well 13 endocannabinoids and related compounds, including the following: anandamide (AEA), 2-arachidonoyl glycerol (2-AG), arachidonic acid (AA), N-arachidonoyl dopamine (NADA), and 2-arachidonic glycerol ether (2-AGe), palmitoyl ethanolamide (PEA), docosatetraenoyl ethanolamide (DEA), oleoyl ethanolamide (OEA), stearoyl ethanolamide (SEA), alpha-linolenoyl ethanolamide (aLEA), gamma-linolenoyl ethanolamide (gLEA), N-arachidonoyl-L-serine (ARA-S), and N-arachidonoyl taurine (NAT). A validated ultra-high pressure liquid chromatography-mass spectrometry (UH-LCMS) method was used to quantify the analytes [32]; details of the analysis are provided in the Supplementary Materials.

Stool samples were collected prior to each dosing session in ethanol tubes by each volunteer at home and posted to the Oxford Centre for Microbiome Studies where they were stored at −80 °C. Details of the analysis are provided in the Supplementary Materials.

### 2.9. Safety and Tolerability

Adverse events were assessed by a medical doctor at the time of each blood draw, and at the final follow-up visit, 7–14 days after the second experiment. All adverse events were recorded. Treatment-emergent adverse events included any adverse event which occurred after drug administration. Treatment-related adverse events included any adverse event that was determined by clinicians to be at least ‘remotely’ related to the study drug. Vital signs (heart rate, systolic and diastolic blood pressure, oxygen saturation, respiratory rate, and temperature) were recorded pre-dose and 1, 2, 4, 8, 24, and 48 h post-dose. Blood samples for LFTs, U&Es and FBC, and a urine sample for urinalysis, were collected at 4 h post-dose.

The Gastrointestinal Symptom Rating Scale (GSRS) was used to assess the subjective severity of reflux, abdominal pain, indigestion, diarrhoea and constipation [33]. Symptom severity was rated 1–7, with a rating of 1 corresponding to ‘No discomfort at all’, and 7 corresponding to ‘very severe discomfort’. In the present study, the scale was adapted to assess symptoms over the past 24 h (as opposed to the past week). Participants completed the scale prior to drug administration and at 24 and 48 h post-drug administration.

### 2.10. Statistical Analysis

The target sample size was 14, satisfying EMA recommendations for minimum sample size (n = 12) in pharmacokinetic studies [34]. The plasma concentration-time data was subject to non-compartmental pharmacokinetic analysis. The primary outcome was the difference in AUC_∞_ for a single dose of oral CBD between the lipid and powder formulations in the fasting state. Secondary outcomes included the following: area under the concentration–time curve from time zero to 48 h (AUC_0–48_); maximum plasma concentration (C_max_); time at which the maximum plasma concentration was reached after administration of the drug (T_max_); and plasma half-life (t_½_).

The log-transformed AUC and C_max_ for each formulation were entered into a linear mixed model to account for the repeated measures and between subject conditions. Linear contrasts representing the difference between conditions were expressed as the ratio of geometric means, with a 95% CI and inference based on *p* < 0.05. The Wilcoxon signed-rank test was used to produce the *p*-value for comparing the geometric means of pharmacokinetic concentrations at each time point.

The incidence of adverse events was compared using McNemar’s test. To assess gastrointestinal symptoms measured by the GSRS scale, the mean scores for each symptom cluster and the mean total score were compared. Because these scores were not normally distributed, they were compared using the Wilcoxon signed-rank test. To assess vital signs, the mean score at each timepoint was compared using a paired sample *t*-test. For the safety, tolerability and adverse effects, no correction multiple comparisons was applied to ensure that all potential effects were reported.

The endocannabinoid plasma concentrations were baseline corrected, then the AUC_0–48_ values for the two formulations were compared using paired *t*-tests and paired Wilcoxon signed-rank tests, depending on whether data followed a normal distribution. The correlations between the AUC_0–48_ for CBD and the AUC_0–48_ for each endocannabinoid were examined using Spearman’s rank correlation, as the plasma exposure of CBD was not normally distributed. Correlations were corrected for multiple comparisons using the Benjamini–Hochberg method.

Microbiome data (including Amplicon Sequence Variance (ASVs), secondary bile acids, and Copies Per Million (CPM) abundances) were used in linear regression analyses, stratified by arm, with CBD AUC_0–48_ as the outcome. *p* values were adjusted for multiple testing using the Benjamini–Hochberg false discovery rate method across all analyses.

All analyses were per protocol and were performed using SAS version 9.4 and R version 4.2.2. To calculate the geometric means and AUCs, we used the DescTools (version 0.99.4) and bayestestR (version 0.13.0) packages. For microbiome analyses, we used the ‘phyloseq’ and ‘microbiome’ packages.

### 2.11. Ethics

The trial was conducted in accordance with Good Clinical Practice and the Declaration of Helsinki. It was approved by Brent Research Ethics Committee (Ref: 22/LO/0047), the UK Health Research Authority and Medicines and Healthcare products Regulatory Agency. Written informed consent was obtained from each subject before any trial-related procedures were performed.

## 3. Materials and Methods—Study 2

### 3.1. Study Drugs

Both formulations contained naturally derived CBD (>99% pure; BSPG Laboratories, Sandwich, Kent, UK). The lipid matrix formulation contained Maisine^®^ CC, Gelucire^®^ 43/01, and polyethylene glycol 400. The aqueous formulation comprised CBD powder dissolved in water.

### 3.2. Biological Tissue Preparation

Fourteen male CD1 mice were prepared by Charles River Laboratories, Edinburgh, UK (Test Facility Study No. 190318). Lipid or aqueous CBD (n = 6 per group) at 25 mg/kg was administered via oral gavage twice daily for 2.5 days [35]. After the fifth dose, one mouse was euthanised at each of the following timepoints: 0 min (immediately post-dose), 30 min, 1 h, 2 h, 4 h and 8 h post-drug administration. Two mice were administered vehicle only (1 lipid vehicle; 1 water vehicle) before being euthanised at 8 h post-dose on day 3.

### 3.3. Plasma PK Profiling with Liquid Chromatography-Mass Spectrometry (LC-MS/MS)

Immediately prior to euthanasia, plasma samples were collected under anaesthesia via the orbital sinus. A 1 mL sample of blood was collected with anticoagulant and then centrifuged (3000 rpm for 10 min at 4 °C). Plasma was aliquoted into cryovials and stored at −80 °C. High-performance LC-MS/MS was performed at Imabiotech (Loos, France) to determine the plasma concentration of CBD. LC-MS/MS could not be performed for the 7-hydroxy and 7-carboxy metabolites of CBD as it was not possible to obtain acceptable calibration curves.

### 3.4. Preparation of Whole-Body Sections for Biodistribution Analysis

Whole-body carcasses were stored at −80 °C until shipment to Imabiotech. A cryostat was used to obtain 20 μm whole-body tissue sections, which were then placed on Indium Tin Oxide slides. Sagittal sections were made through the left side of the animal and included the brain, stomach, intestines, liver, lungs, kidney, muscles, heart, eye and fat. The slides were dried in a desiccator for 15 min to assist analyte desorption and ionization, and the samples were sprayed with a 1,5-diaminonaphthalene matrix prepared in acetonitrile/water that had been spiked with an internal standard, CBD-D3.

### 3.5. Whole-Body Mass Spectrometry Imaging (MSI) Using Matrix-Assisted Laser Desorption/Ionization (MALDI)

Images were obtained using a 7T MALDI-FTICR (SolariX, Bruker, Billerica, MA, USA) in CASI negative mode (315 Da with 40 Da window) at a 350 μm spatial resolution to image CBD and the CBD-D3 internal standard. An optical image of the slides was acquired to enable the synchronisation of the tissue sections with the target of the laser.

### 3.6. Ethics

The study was conducted under UK Home Office Project Licence No. PP9376768 (Charles River Laboratories (UK)), Pharmacokinetics of Pharmaceuticals, Protocol Reference Number 1.

## 4. Results—Study 1

In total, 14 participants completed the study: seven males and seven females, with a mean age of 26.7 (SD: 4.4). The participants were of white (n = 10; 71.4%) or Asian (n = 4, 28.6%) ethnicity. The mean BMI was 21.9 kg/m^2^ (SD: 1.81). All participants reported fasting prior to both sessions. For all sessions that were tested (n = 21; 13 participants), fasting status was confirmed by the concentration of serum bile acids being within the normal fasting reference range (1–9 μmol/L [31,36]. The mean serum bile acid concentration across participants was 3.2 μmol/L.

### 4.1. Pharmacokinetics of CBD and Metabolites

**Figure 1:**
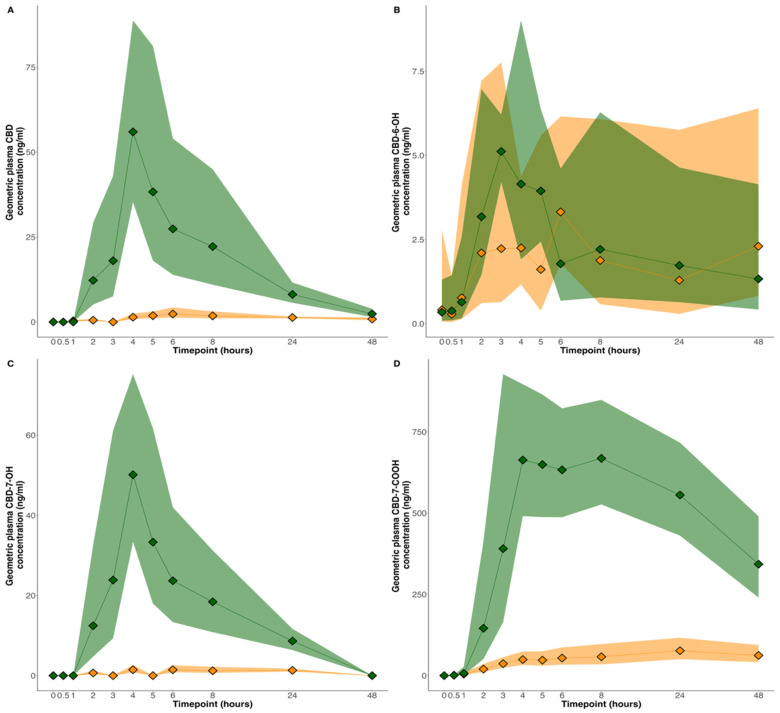
Pharmacokinetic profile of CBD and its metabolites for the lipid (green) and powder (orange) formulations. (**A**) CBD; (**B**) 6-OH-CBD; (**C**) 7-OH-CBD; and (**D**) 7-COOH-CBD. Data presented are geometric means with 95% confidence intervals (shaded area).

The geometric mean plasma concentration–time profile of CBD and its metabolites is presented in Table 1 and Figure 1. The AUC_0–48_ was approximately 9× higher and the C_max_ was 24× higher with the lipid compared to the powder formulation. Wilcoxon signed-rank sum tests demonstrated that the AUC_inf_, AUC_0–48_ and C_max_ were higher for CBD and for two of its metabolites (7-COOH-CBD and 7-OH-CBD) when the lipid formulation was compared with the powder formulation (*p* < 0.05). The t_max_ was 4 h for the lipid formulation and 6 h for the powder formulation. THC and THC-COOH were not detected in any of the blood samples.

### 4.2. Safety, Tolerability and Adverse Events

No participant withdrew from the study and there were no serious adverse events. The blood and urine samples collected 4 h post-treatment demonstrated no clinically relevant abnormalities. There were no statistically significant differences in vital signs at any timepoint, apart from the diastolic blood pressure, which was 8 mm Hg higher at the pre-dose timepoint in the powder arm (paired sample *t*-test; *p* = 0.04).

Adverse events are reported in Table 2. Treatment with the lipid formulation was associated with more reports of somnolence (seven vs. two; *p* = 0.025). Treatment-emergent and treatment-related adverse events are reported in the Supplementary Table S3.

At 24 h post-treatment, treatment with the lipid formulation was associated with a mean rating of 1.43 for diarrhoea (SD: 0.89), compared to 1.00 (SD: 0.0) for the powder formulation, as measured by GSRS (Supplementary Table S4). A rating of 1.43 is lower than that corresponding to ‘Minor discomfort’ (2), but higher than that for ‘No discomfort at all’ (1). At 48 h post-treatment, treatment with the lipid formulation was associated with a rating of 1.09 (SD: 0.18) for gastrointestinal symptoms overall, versus 1.02 (SD: 0.04) for the powder formulation.

### 4.3. Plasma Endocannabinoids

**Figure 2:**
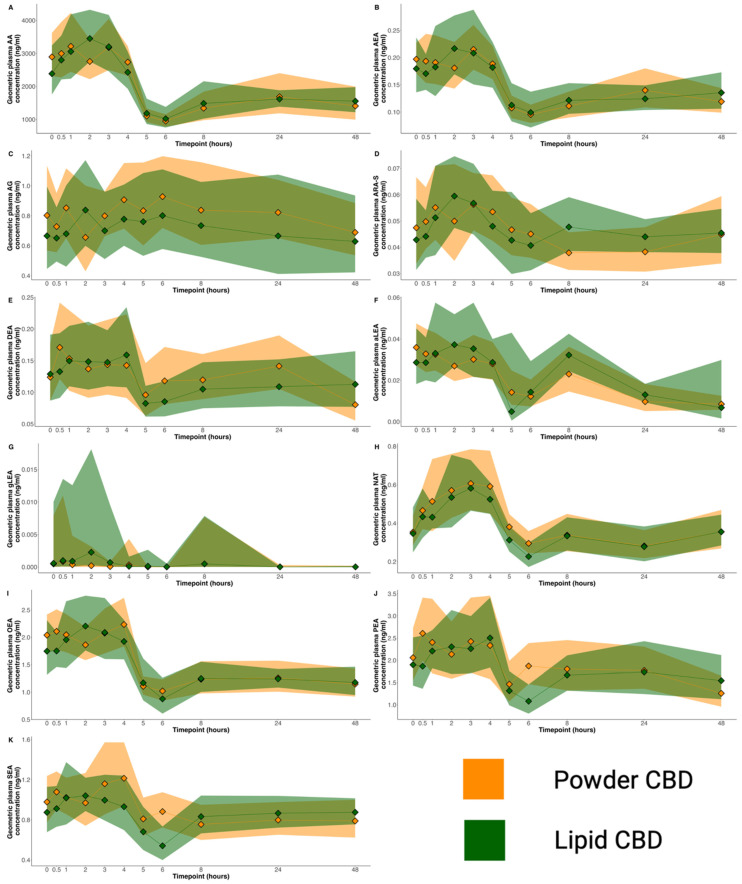
Plasma concentration–time profile of 11 endocannabinoids and related compounds after treatment with the lipid formulation (green) and powder formulation (orange). (**A**) Arachidonic acid; (**B**) Anandamide; (**C**) 2-arachidonoyl glycerol; (**D**) N-arachidonoyl-L-serine; (**E**) Docosatetraenoyl ethanolamide; (**F**) Alpha-linolenoyl ethanolamide; (**G**) Gamma-linolenoyl ethanolamide; (**H**) N-arachidonoyl taurine; (**I**) Oleoyl ethanolamide; (**J**) Palmitoyl ethanolamide; (**K**) Stearoyl ethanolamide. Data presented are geometric means with 95% confidence intervals (shaded areas).

The plasma concentration of 13 endocannabinoids and related compounds was analysed at each timepoint (Table 3 and Figure 2). NADA and 2-AGe were not detected in any sample. There were no statistically significant differences in AUC_0–48_ or C_max_ between the two formulations for any endocannabinoid or related compound. There were statistically significant correlations between the CBD AUC_0–48_ and the AUC_0–48_ of AA (r = 0.43, *p* = 0.02) and ARA-S (r = 0.43, *p* = 0.02) (Table 3). Since these were exploratory analyses, we also applied a multiple comparison correction. None of the correlations were statistically significant after correction (AA, *p* = 0.12; ARA-S, *p* = 0.12).

## 5. Microbiome

### 5.1. Lipid Formulation

Ten participants provided stool samples in advance of both experimental visits for microbiome analysis. A greater absorption of the lipid formulation of CBD, as measured using the plasma AUC_0–48_, was associated with lower levels of the genus *Helicobacter* (β = −1.5; SE = 0.4; *p*_corr_ = 0.013), *Ruminoclostridium* (β = −2.9; SE = 0.7; *p* = 0.012) and *Mailhella* (β = −2.8; SE = 0.7; *p*_corr_ = 0.049), and higher concentrations of the genus *Lentilactobacillus* (β = 1.2; SE = 0.3; *p*_corr_ = 0.049). There was no association with any of the other microbiome measures (Shannon index, beta-diversity indices, Kegg metabolic pathways, or secondary bile acids).

### 5.2. Powder Formulation

A greater absorption of the powder formulation of CBD, as measured using the plasma AUC_0–48_, was associated with higher concentrations of *Lachnoclostridium* (β = 27.2; SE = 7.3; *p*_corr_ = 0.017), *Paraprevotella* (β = 6.4; SE = 1.9; *p*_corr_ = 0.032), *Methanosphera* (β = 5.3; SE = 1.4; *p*_corr_ = 0.017), *Oxalobacter* (β = 4.7; SE = 1.1; *p*_corr_ = 0.001), and *Kallipyga* (β = 6.6; SE = 1.8; *p*_corr_ < 0.001), and lower concentrations of *Kyptococcus* (β = −7.8; SE = 2.3; *p*_corr_ = 0.027) and *Xylanimonas* (β = −6.5; SE = 1.9; *p*_corr_ = 0.027). A greater absorption of CBD was also associated with the concentration of three secondary bile acids: taurodeoxycholic acid (TDCA; β = 16.8; SE = 4.2; *p*_corr_ = 0.001), taurolithocholic acid (TLCA; β = 16.8; SE = 4.5; *p*_corr_ = 0.002) and glycodeoxycholic acid (GDC; β = 16.6; SE = 6.1; *p*_corr_ = 0.033). There was no association with any of the other microbiome measures (Shannon index, beta-diversity indices, Kegg metabolic pathways).

## 6. Results—Study 2

### 6.1. Plasma Concentration of CBD Measured by LC-MS/MS

#### 6.1.1. Lipid Formulation

The peak concentration (C_max_) of CBD was 198 ng/mL at 1-h post-drug administration (T_max_). The AUC_0–8_ was 335 ng/mL·h and the AUC_∞_ was 381 ng/mL. The half-life of CBD was 2.4 h, and the oral clearance (CL/F) was 66 L/kg/h. The volume of distribution was 230 L/kg.

#### 6.1.2. Aqueous Formulation

Pharmacokinetic parameters could not be generated as the plasma concentrations were either below the limit of quantification or CBD was undetectable (Table 4). CBD was not detected in the plasma of either the lipid vehicle-only control or the aqueous vehicle-only control.

### 6.2. Biodistribution of CBD

#### 6.2.1. Lipid Formulation

**Figure 3:**
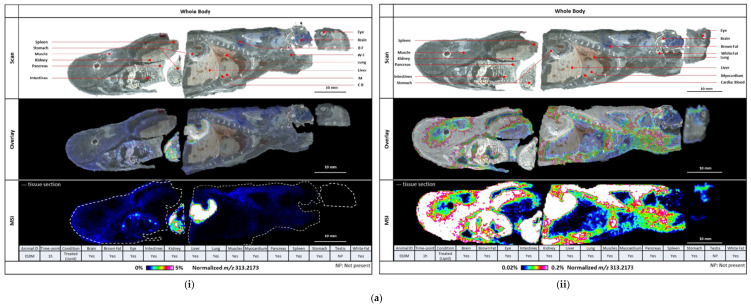

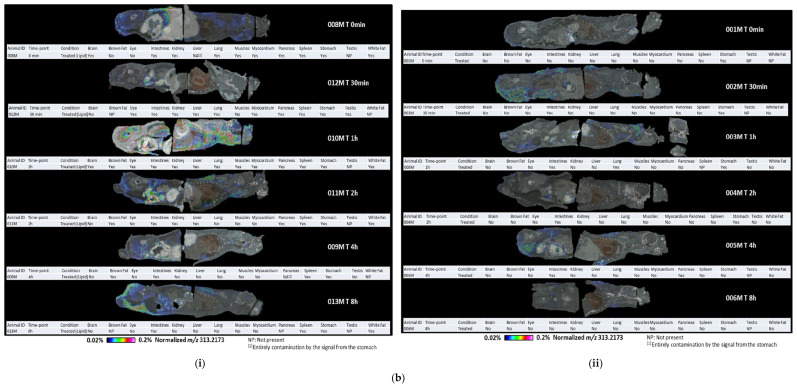
(**a**). Sagittal sections showing whole-body biodistribution of the lipid formulation of CBD at 1 h (plasma T_max_) (**i**) Colour scale: 0% to 5%; (**ii**) Colour scale: 0.02% to 0.2%. (**b**) Sagittal sections showing whole-body biodistribution of CBD at several timepoints after treatment with (**i**) the lipid formulation and (**ii**) the aqueous formulation.

CBD was detected in the brain, eye, intestines, kidney, liver, lung, muscle, myocardium, pancreas, spleen stomach, and adipose tissues (Table 4; Figure 3). In the testis, it was only detected at levels below the level of quantification. For most organs, the T_max_ was 1 h post-drug administration (Table 4). The peak concentration was between 1.6 and 3.4 μg/g of tissue for all organs, apart from the gastrointestinal tract (78.0 μg/g; >ULOQ) and brown and white fat tissues (15.8 and 9.0 μg/g, respectively).

#### 6.2.2. Aqueous Formulation

CBD was detected in the stomach and intestines at several timepoints, but was not consistently evident in other tissues. The T_max_ (1 h) and C_max_ (59.5 μg/g of tissue) in the stomach with the aqueous formulation treatment differed markedly from those for the lipid formulation (T_max_ = 0 min; C_max_ = 2514 μg/g of tissue, extrapolated value).

## 7. Discussion

Our main findings were that in human volunteers in the fasting state, a novel lipid-matrix formulation of oral CBD achieved a plasma exposure nine times higher than with a lipid-free formulation, and a C_max_ that was 24 times higher. The absorption of CBD in the powder formulation was significantly associated with a number of microbiome variables that were less evident when CBD was administered in the lipid formulation. Both formulations were well tolerated. The lipid formulation was associated with more reports of somnolence, but most of these were determined to not be treatment related. A parallel study in rodents revealed similar pharmacokinetic differences between the two formulations, and a higher concentration of CBD in target organs.

The only formulation of CBD licensed for clinical use is Epidiolex, in which CBD is dissolved in sesame oil. A number of previous studies have investigated the pharmacokinetics of Epidiolex in healthy volunteers in the fasting state using single doses comparable to those used in the present human study. Schoedel and colleagues reported that a 750 mg dose was associated with a C_max_ of 336.2 ng/mL (CV%: 46.7) and an AUC_0–24_ of 1586.7 ng·h/mL (CV%: 52.0) [37]. Taylor and colleagues found that a 1500 mg dose yielded a C_max_ of 292.4 ng/mL (CV%: 87.9) and an AUC_0–48_ of 1517 ng·h/mL (CV%: 78.2) [13]. Crockett and colleagues used a 750 mg dose of Epidiolex in the fasting state, and reported a C_max_ of 187 ng/mL (CV%: 52.2) and an AUC_t_ of 1077 ng·h/mL (CV%: 49.6) [14]. However, comparisons across pharmacokinetic studies should be made with caution, as differences in the participant samples and testing conditions may influence the results, especially when the sample sizes are small. In one of the few studies that has compared different lipid formulations head to head, Izgelov and colleagues found that a single dose (90 mg) of a novel SNEDDS formulation of CBD in fasting healthy volunteers achieved plasma concentrations that were similar to a sesame oil formulation intended to resemble the formulation used in Epidiolex [16].

In our human study, there were large fluctuations in the concentrations of several analytes (e.g., arachidonic acid, anandamide, N-arachidonoyl taurine, DEA, OEA and PEA) following the administration of CBD. Endocannabinoids are known to be affected by factors such as circadian rhythm, food intake, exercise, and stress [38], and the largest fluctuations were between 4 and 5 h post-drug administration, when participants were allowed to eat for the first time following an extended fast. To investigative CBD’s mechanism of action, we conducted exploratory correlation analyses between CBD and the endocannabinoids. However, there were no statistically significant correlations after correction for multiple comparisons.

The data from the human stool samples suggest a potential link between the gut microbiome and the absorption of CBD. Higher levels of faecal secondary bile acids were strongly associated with an increased absorption of CBD in the powder, but not the lipid formulation. Secondary bile acids are bacterial byproducts that facilitate the formation of micelles, which enhance the absorption of highly lipophilic drugs like CBD [27]. The lipids coating the CBD in the lipid formulation may reduce the influence of these bacterial bile acids on absorption. Because the concentration of the latter varies as a function of inter-individual differences in the gut microbiome, the lipid formulation may thereby reduce inter-subject variability in the absorption of oral CBD.

The effects of different CBD formulations on its oral absorption have been extensively investigated in preclinical models. Feng and colleagues administered seven different natural CBD oil-based formulations (sesame, soybean, olive, sunflower, coconut, peanut oil, and a lipid-free formulation containing propylene glycol, ethanol and water) to rats [39]. All of the oil formulations increased the plasma AUC when compared to a lipid-free formulation, except for those involving sunflower and coconut oil. The authors attributed the poor performance of these oils to their relatively high medium-chain triglyceride content (as opposed to long-chain triglycerides). The highest AUCs were achieved with sesame and olive oils, both of which are particularly rich in oleic acid and linoleic acid, unsaturated long-chain (18-carbon) fatty acids that are believed to stimulate chylomicron formation and promote intestinal absorption via the lymphatic system [40]. The same group developed two novel formulations combining sesame oil with either medium-chain triglycerides, or with medium-chain triglycerides, a solubiliser and surfactants, which were expected to enhance emulsification and micellar solubilization [41]. However, when tested in a rat model, the systemic bioavailability and lymphatic transport of all these formulations were inferior to those with a pure sesame oil formulation. Similar results were found in a study which investigated two novel SNEDDS formulations, which increased CBD exposure by 2–3 fold compared to a medium-chain triglyceride formulation, but were not associated with a greater bioavailability than Epidiolex [42]. Other approaches for improving the bioavailability of CBD have involved formulations using enzyme inhibitors, gastro-retentive formulations, and cyclodextrins [43,44,45].

In our rodent study, we demonstrated the feasibility of using MALDI to measure the tissue biodistribution of CBD. CBD administered in the lipid formulation was detected in all organs at similar concentrations, apart from the gastrointestinal tract and adipose tissues, where the concentrations were considerably higher. For the aqueous formulation, CBD was not detected in the liver or any target organ; it was present in the stomach and intestines, but this would be expected following oral administration. These findings and the plasma data suggest that very little of the aqueous formulation was absorbed. The small sample of the rodent study limits the further interpretation of its results.

The strengths of the present study include the use of a lipid technology that has already been shown to enhance the oral bioavailability of ibuprofen and has been incorporated into an approved formulation [23]. To our knowledge, this is the first study to explore the influence of the gut microbiome on CBD absorption. In our human experiments, the fasting status of the participants was confirmed by measuring serum bile acid levels. In our rodent study, while it was clear that the lipid formulation had superior bioavailability, absorption of the aqueous formulation was too low to allow a formal comparison of the PK levels and biodistribution patterns. In addition, it was not possible to produce acceptable calibration curves to permit an analysis of CBD’s metabolites in plasma. These data may have clarified the extent to which the greater delivery of CBD to putative target organs with the lipid formulation was related to increased absorption versus reduced first-pass metabolism. A major limitation of the rodent study is the small sample size, with only n = 1 animal per timepoint.

In summary, we found that a novel lipid formulation improved the bioavailability of oral CBD, while reducing the influence of the gut microbiome on its absorption. Future studies could directly compare this formulation with Epidiolex and examine the impact of food on its bioavailability.

## Figures and Tables

**Table 1:** Pharmacokinetic parameters of CBD and its metabolites.

Pharmacokinetic Parameter (Unit)	Novel Lipid Formulation	Powder Formulation	*p* Value
CBD			
AUC_inf_ (ng·h/mL) ^a^	675.0 (103.1)	134.8 (88.7) *	0.001
AUC_0–48_ (ng·h/mL) ^a^	611.4 (104.6)	66.8 (50.7)	<0.001
C_max_ (ng/mL) ^a^	73.0 (149.8)	3.1 (103.6)	<0.001
T_max_ (h) ^b^	4 (2–8)	6.0 (3–48)	na
t_½_ (h) ^c^	13.7 (42.9)	51.8 (111.2) *	na
6-OH-CBD			
AUC_inf_ (ng·h/mL) ^a^	173.3 (497.0)	136.5 (196.1)	0.588
AUC_0–48_ (ng·h/mL) ^a^	112.6 (211.8)	96.0 (245.0)	0.828
C_max_ (ng/mL) ^a^	8.2 (69.0)	6.5 (110.5)	0.779
T_max_ (h) ^b^	4 (0.5–48)	6 (1–48)	na
t_½_ (h) ^c^	54.0 (103.9)	34.8 (52.0)	na
7-OH-CBD			
AUC_inf_ (ng·h/mL) ^a^	692.5 (83.2)	130.6 (232.9) *	0.016
AUC_0–48_ (ng·h/mL) ^a^	568.4 (76.7)	56.1 (72.2) *	<0.001
C_max_ (ng/mL) ^a^	63.7 (87.9)	2.4 (101.2) *	<0.001
T_max_ (h) ^b^	4 (2–5)	6 (2–48) *	na
t_½_ (h) ^c^	20.5 (77.3)	111.1 (157.4) *	na
7-COOH-CBD			
AUC_inf_ (ng·h/mL) ^a^	73,404.3 (188.3)	9924.9 (140.3) *	0.011
AUC_0–48_ (ng·h/mL) ^a^	24,523.0 (43.4)	3142.6 (81.1)	<0.001
C_max_ (ng/mL) ^a^	823.0 (36.6)	91.9 (85.9)	<0.001
T_max_ (h) ^b^	4 (2–8)	24 (3–48)	na
t_½_ (h) ^c^	259.5 (216.5)	68.1 (48.0) *	na

^a^ Geometric mean (geometric coefficient of variation [CV%]); ^b^ Median (range); ^c^ Arithmetic mean (arithmetic CV%). * In some instances, where metabolites were not detected or continued to increase in concentration until the final timepoint, a restricted sample was used to calculate parameters. This issue affected the powder arm for the following metabolites: CBD, n = 11; 6-OH-CBD n = 6–9, 7-OH-CBD, n = 9; 7-COOH-CBD, n = 5. na: not analysed.

**Table 2:** Adverse events.

Adverse Event	Lipid Formulation (n = 14)	Powder CBD Formulation (n = 14)	*p* Value
Any adverse event	11 (78.6%)	8 (57.1%)	0.26
Gastrointestinal disorders	5 (35.7%)	2 (14.3%)	0.18
Diarrhoea	2 (14.3%)	0 (0.0%)	0.16
Nausea	1 (7.1%)	1 (7.1%)	1.00
Vomiting	0 (0.0%)	1 (7.1%)	0.32
Flatulence	1 (7.1%)	1 (7.1%)	1.00
Abdominal distension	1 (7.1%)	0 (0.0%)	0.32
Abdominal pain	1 (7.1%)	0 (0.0%)	0.32
Neuropsychiatric disorders	9 (64.3%)	8 (57.1%)	0.71
Somnolence	7 (50.0%	2 (14.3%)	0.025
Headache	2 (14.3%)	3 (21.4%)	0.56
Dizziness	1 (7.1%)	1 (7.1%)	1.00
Relaxed	0 (0.0%)	1 (7.1%)	0.32
High	0 (0.0%)	1 (7.1%)	0.32
Anxiety	1 (7.1%)	0 (0.0%)	0.32
Vasovagal	1 (7.1%)	1 (7.1%)	1.00

**Table 3:** The maximum plasma concentration, exposure, and correlation with CBD exposure of eleven endocannabinoids and related compounds.

Compound	Lipid Formulation		Powder Formulation		Both Formulations	
	C_max_ (ng/mL)	AUC_0–48_ (ng·h/mL)	C_max_ (ng/mL)	AUC_0–48_ (ng·h/mL)	CBD AUC_0–48_ × AUC_0–48_ Correlation	Corrected *p* Value
AEA	0.29 (0.10)	−2.63 (2.81)	0.28 (0.08)	−2.67 (1.56)	r = −0.22, *p* = 0.25	0.46
2-AG	1.37 (0.81)	−0.59 (23.16)	1.40 (0.67)	−3.34 (22.60)	r = −0.12, *p* = 0.56	0.64
AA	4116.21 (1562.93)	−39,817.96 (46,909.77)	4309.05 (1114.65)	−54,629.54 (33,436.99)	r = −0.43, *p* = 0.02	0.12
PEA	4.25 (1.73)	−4.25 (65.80)	5.53 (3.10)	−17.85 (54.58)	r = −0.12, *p* = 0.53	0.64
DEA	0.30 (0.16)	−1.52 (6.09)	0.39 (0.24)	−0.10 (4.72)	r = −0.11, *p* = 0.58	0.64
OEA	2.77 (0.84)	−25.43 (24.65)	3.02 (0.74)	−35.21 (31.92)	r = −0.23, *p* = 0.24	0.46
SEA	1.66 (0.49)	−1.19 (19.40)	1.79 (0.47)	−8.74 (15.34)	r = −0.34, *p* = 0.08	0.29
aLEA	0.07 (0.03)	−0.70 (0.8)	0.06 (0.03)	−1.02 (0.81)	r = −0.28, *p* = 0.15	0.41
gLEA	0.04 (0.02)	−0.56 (0.91)	0.03 (0.02)	−0.47 (1.02)	r = −0.14, *p* = 0.49	0.64
ARA-S	0.09 (0.03)	−0.03 (1.05)	0.09 (0.02)	−0.46 (1.02)	r = −0.43, *p* = 0.02	0.12
NAT	0.90 (0.65)	−1.84 (8.33)	0.98 (0.32)	−0.36 (6.28)	r = −0.03, *p* = 0.87	0.87

Geometric mean (geometric coefficient of variation [CV%]). AEA: anandamide; 2-AG: 2-arachidonoyl glycerol; AA: arachidonic acid; PEA: palmitoyl ethanolamide; DEA: docosatetraenoyl ethanolamide; OEA: oleoyl ethanolamide; SEA: stearoyl ethanolamide; aLEA: alpha-linolenoyl ethanolamide; gLEA: gamma-linolenoyl ethanolamide; ARA-S: N-arachidonoyl-L-serine; NAT: N-arachidonoyl taurine.

**Table 4:** Plasma concentration (ng/mL) and MALDI quantification of CBD (μg/g of tissue) in whole-body sections.

**Lipid Arm**															
**Timepoint**	**Plasma**	**Brain**	**Brown Fat**	**Eye**	**Intestines**	**Kidney**	**Liver**	**Lung**	**Muscle**	**Myocardium**	**Pancreas**	**Spleen**	**Stomach**	**Testis**	**White Fat**
0	1.8	3.1	ND	ND	46.9	2.1	NA *	1.2	2.8	1.2	1.5	0.6	2514 (ULOQ)	NP	2.5
0.5	4.3	ND	NP	2.4	78.0 (ULOQ)	3.0	1.6	BLOQ	ND	BLOQ	1.4	3.7	1781 (ULOQ)	BLOQ	NP
1	198.3	2.3	15.8	0.7 (BLOQ)	19.4	2.4	1.6	2.6	3.4	2.5	1.8	2.9	127.4 (ULOQ)	NP	8.5
2	22.6	ND	14.8	ND	12.1	1.7	1.6	ND	ND	ND	2.7	1.6	392.7 (ULOQ)	NP	9.0
4	41.4	ND	6.5	ND	24.8	ND	ND	ND	ND	ND	NA *	2.6	1076.1 (ULOQ)	ND	NP
8	12.9	ND	NP	ND	4.3	ND	ND	ND	ND	ND	NP	1.0	9.0	NP	3.4
**Aqueous Arm**															
**Timepoint**	**Plasma**	**Brain**	**Brown Fat**	**Eye**	**Intestines**	**Kidney**	**Liver**	**Lung**	**Muscle**	**Myocardium**	**Pancreas**	**Spleen**	**Stomach**	**Testis**	**White Fat**
0	BLOQ (0.6)	ND	ND	ND	ND	ND	ND	ND	ND	ND	ND	ND	9.1	NP	NP
0.5	ND	ND	ND	ND	2.4	ND	ND	ND	ND	ND	ND	ND	5.2	NP	ND
1	BLOQ (0.5)	ND	ND	ND	0.9	ND	ND	0.4 (BLOQ)	ND	ND	ND	NP	59.5	ND	ND
2	ND	ND	ND	ND	3.2	ND	ND	ND	ND	ND	ND	ND	43.2	ND	ND
4	ND	ND	ND	ND	19.9 (ULOQ)	ND	ND	ND	ND	ND	1.6	ND	ND	NP	ND
8	ND	ND	ND	ND	ND	ND	ND	ND	ND	ND	ND	ND	ND	NP	ND

ND: Not detected; NP: Not present; BLOQ: Below Limit of Quantification; ULOQ: Upper Limit of Quantification. * Contaminated by the high signal from the surrounding stomach (delocalization).

## Data Availability

The data presented in this study are available on reasonable request from the corresponding author.

## Supplementary Materials

The following supporting information can be downloaded at: https://www.mdpi.com/article/10.3390/pharmaceutics16121537/s1, Table S1: Internal standards used in endocannabinoid analyses; Table S2: Internal standards used in bile acid analyses; Table S3: Treatment-emergent and treatment-related adverse events; Table S4: Gastrointestinal symptoms measured by the GSRS. References cited in the Supplementary Materials include [32,46,47].
