# Supplementary material for: Novel Lipid Formulation Increases Absorption of Oral Cannabidiol (CBD)
Source: Pharmaceutics. 2024 Dec 1;16(12):1537. doi: 10.3390/pharmaceutics16121537 (PMC11728838; doi:10.3390/pharmaceutics16121537)
Supplement: Supplementary file 1 [file pharmaceutics-16-01537-s001.zip › pharmaceutics-3325283-supplementary.pdf]

Supplementary Materials

1. Methods
    a. Blood processing and analysis
        i. Table S1. Internal standards used in endocannabinoid analyses
        ii. Table S2. Internal standards used in bile acid analyses
    b. Stool sampling, processing, and analysis
    c. Plasma endocannabinoids – statistical analysis

2. Results
    a. Adverse effects
        i. Table S3. Treatment-emergent and treatment-related adverse events
        ii. Table S4. Gastrointestinal symptoms measured by the GSRS

Blood processing and analysis

A validated ultra-high pressure liquid chromatography-mass spectrometry (UH-LCMS) method was used to quantify the analytes [1]. An aliquot (200 µL) of sample was added to 400 µL of crash solvent (acetonitrile with 0.1 % formic acid and the internal standards listed in Table S1). The samples were vortexed and left to settle in -20°C for 30 min. After that, the samples were filtered through protein precipitation filter plates and evaporated under a gentle nitrogen stream at 35 °C. The samples were reconstituted in 50 µL solvent (40 % water, 30 % ACN and 30 % IPA).

The LC separation was performed on a Sciex exion AD 30 (AB Sciex Inc., Framingham, MA) LC system consisting of a binary pump, an autosampler set to 15 °C and a column oven set to 40 °C. A waters XBridge BEH C18 (2.5µm, 2.1x150mm) column with a precolumn with the same material was used. Eluent A was 1mM ammonium acetate and 0.1 % formic acid in water and eluent B was 1mM ammonium acetate and 0.1 % formic acid in 50:50 acetonitrile:isopropanol. The gradient was held at 60 % B for 0.3 minutes, and then linearly increased to 100 % B over 5 minutes. The gradient was held at 100 % B for 2.5 minutes, then decreased to 60 % B over 0.1 minutes and the column was re-equilibrated for 3.1 minutes. The flow rate was 0.4 mL/min and the injection volume was 10 µL.

The mass spectrometer used was a Sciex 7500 QTrap mass spectrometer. The full list of the parameters used can be found in Table S1. The ion source gas1 and 2 were 40 and 70 psi respectively. The curtain gas was 40 psi, the CAD gas was 10 and the temperature was 400 °C. The spray voltage was 1500 V. Data processing was performed on Sciex OS.

Bile acids were analysed using a quantitative targeted method [2]. A 20 µL aliquot of plasma or faecal sample was mixed with 200 µL of a crash solution comprising methanol spiked with internal standards and filtered through a protein precipitation filter. The filtrate was transferred to a vial and dried under a stream of nitrogen at 45 °C. The dried sample was resuspended in 20 µL of methanol in water (4:6 v/v) and stored at -80 °C until analysis.

Bile acid analyses were performed on a Shimadzu LC40 system (Shimadzu, Kyoto, Japan) coupled to a Sciex QTrap 5500 mass spectrometer (Sciex) with an atmospheric electrospray interface. Aliquots of 5 µL of samples were injected into the ACQUITY Premier HSS T3

column (2.1 x 100 mm, 1.8 µm) column (Waters Corporation). The eluent system consisted of (A) Water/Metanol (7:3) with 0.1% Formic acid (v/v) and (B) Methanol with 0.1% Formic acid (v/v). The gradient was programmed as follows: 0–0.5 min, 5% B; 0.5–3.5 min, gradient increasing to 60% B; 3.5–11 min, gradient increasing to 100% solvent B; 11–16 min, 100% B, 16-17 min 5 % B, flow rate 0.4 mL/min. The source conditions were curtain gas 35, Collision gas 12, IonSpray voltage -4500 V, temperature 650 °C, Ion source gas 1 and 2 40. The list of analytes and their mass spectrometric parameters can be found in Table S2.

The samples were quantified using calibration curves prepared with concentrations between 0.01 and 100 ppb for cannabinoids and endocannabinoids and 0.025 and 600 ppb for bile acids using the internal standard method. Additionally, one blank, one standards sample and one quality control (QC) sample was prepared for every 10 samples. The QC samples were prepared by pooling an aliquot of every sample before extraction, then extracting them the same way as the other samples. The data was pre-processed using SciexOS. The limit of detection was reported as the lowest calibration point with an accuracy between 80 and 120 % and the coefficient of variance for the quality control sample was reported (Table S2).

Supplementary Materials: Novel Lipid Formulation Increases Absorption of Oral Cannabidiol (CBD)

Table S1. Internal standards used in endocannabinoid analyses

Compound name	Abbreviation	Type	Product code	Supplier	Concentration in crash solution (ppb)	Type	Mode	Q1	Q3	Dwell time	EP	CE	CXP	RT	Q0	Limit of detection	Relative standard deviation in pure standard	Relative standard deviation in plasma quality control sample
2-Arachidonoyl glycerol	2-AG	Standard	A8973-5MG	Sigma	NA	Quantifier	Positive	379.3	287.3	10	10	21	15	4	0	0.05	5%	11%
						Qualifier	Positive	379.3	203.2	10	10	21	15	4.01	0			
2-Arachidonoyl glycerol d5	2-AG d5	Internal standard		Sigma	25	Quantifier	Positive	384.3	287.3	50	10	21	15	4	0			
2-Arachidonic glycerol ether/Noladin ether	2-AGe	Standard	SML0473-5MG	Sigma	NA	Quantifier	Positive	365.4	273.2	50	10	20	15	4.29	10			
6-hydroxy-Cannabidiol	6-OH-CBD	Standard	C-045-1ML	Cerillant	NA	Quantifier	Negative	329.3	261.1	50	-10	-27	-14	2.14	10	0.5	4%	85%
7-COOH-Cannabidiol	7-COOH CBD	Standard	C-181-1ML	Cerillant	NA	Quantifier	Negative	343.1	299.1	50	-10	-26	-14	2.25	10	0.1	3%	8%
7-COOH-Cannabidiol-d3	7-COOH-CBD d3	Internal standard	C-224-1ML	Cerillant	2.5	Quantifier	Negative	346.3	302.1	50	-10	-25	-14	2.25	10			
7-hydroxy-Cannabidiol	7-OH-CBD	Standard	C-180-1ML	Cerillant	NA	Quantifier	Negative	329	261.1	50	-10	-28	-14	2.27	10	0.5	3%	7%
7-hydroxy-Cannabidiol-d3	7-OH-CBD d3	Internal standard	C-2243-1ML	Cerillant	2.5	Quantifier	Negative	332.3	264.2	50	-10	-27	-14	2.27	10			
Arachidonic acid	AA	Standard	A3611-100MG	Sigma-Aldrich	NA	Quantifier	Negative	303.3	259.1	5	-10	-20	-14	4.51	-50	3	3%	5%
Arachidonic acid d8	AA d8	Internal standard		Cayman Chemical Company	250	Quantifier	Negative	311.3	267.1	50	-10	-20	-14	4.48	-50			
Arachidonoyl ethanolamide	AEA	Standard	P0359-10MG	Sigma		Quantifier	Positive	348.3	62.1	10	10	32	15	3.86	40	0.05	6%	17%
Arachidonoyl ethanolamide d8	AEA d8	Internal standard	9000552	Cayman Chemical Company	2.5	Quantifier	Positive	355.6	62.1	10	10	30	15	3.84	40			
Alpha-linolenoyl ethanolamide	aLEA	Standard	90215	Cayman Chemical Company	NA	Quantifier	Positive	322.4	62.1	50	10	18	15	3.46	30	0.01	2%	51%
Alpha-linolenoyl ethanolamide-d4	aLEA d4	Internal standard	9001841	Cayman Chemical Company	2.5	Quantifier	Positive	326.4	66	10	10	25	15	3.46	30			
Cannabidiol	CBD	Standard	C-045	Cerillant	NA	Quantifier	Positive	315.3	193	50	10	30	15	3.32	-10	0.01	4%	13%
Cannabidiol d3	CBD d3	Internal standard	C-084-1ML	Cerillant	2.5	Quantifier	Positive	319.3	196	50	10	29	15	3.32	-10			
Docosatetraenoyl ethanolamide	DEA	Standard	90385	Cayman Chemical Company	NA	Quantifier	Positive	376.3	62.1	10	10	22	15	4.36	60	0.01	5%	18%
Gamma-linolenoyl ethanolamide	gLEA	Standard	9001747	Cayman Chemical Company	NA	Quantifier	Positive	322.3	62.1	50	10	19	15	3.57	-10	0.1	2%	130%
						Qualifier	Positive	322.3	261.3	20	10	19	15	3.57	-10			
N-arachidonoyl-L-serine	ARA-S	Standard	10005455	Cayman	NA	Quantifier	Negative	390.3	360.3	50	-10	-26	-14	3.71	-70	0.05	5%	23%
N-arachidonoyl-L-serine d8	ARA-S d8	Internal standard	10007428	Cayman	2.5	Quantifier	Negative	398.6	368.4	10	-10	-27	-14	3.69	-70			
N-arachidonoyl taurine	NAT	Standard	10005537	Cayman Chemical Company	NA	Quantifier	Negative	410.7	80	50	-10	-70	-14	3.17	-100	0.01	18%	58%
Oleoyl ethanolamide	OEA	Standard	O0383-25MG	Sigma	NA	Quantifier	Positive	326.3	62.1	10	10	20	15	4.49	40	0.5	5%	11%
Oleoyl ethanolamide -d4	OEA d4	Internal standard		Cayman Chemical Company	2.5	Quantifier	Positive	330.6	66	25	10	20	15	4.48	40			
Palmitoyl ethanolamide	PEA	Standard	PO359	Sigma	NA	Quantifier	Positive	300.4	62.1	35	10	19	15	4.42	40	0.5	3%	28%
						Qualifier	Positive	300.4	283.1	35	10	19	15	4.42	40			
Stearoyl ethanolamide	SEA	Standard	S8439-5MG	Sigma	NA	Quantifier	Positive	328.4	311.4	85	10	23	15	5.09	60	0.05	3%	9%
Stearoyl ethanolamide-d3	SEA d3	Internal standard	14726	Cayman Chemical Company	2.5	Quantifier	Positive	331.1	62	25	10	41	15	5.07	60			

Table S2. Internal standards used in bile acid analyses

Compound name	Abbreviation	Type	Concentration in crash solution (ppb)	Q1	Q3	RT	EP	CE	CXP	Q0	Limit of detection	Relative standard deviation in pure standard	Relative standard deviation in plasma quality control sample
Taurodeoxycholic acid	TDHCA	Standard		508.3	80	2.81	-10	-102	-14	-80	0.25	5%	22%
Tauro-alpha-muricholic acid	TaMCA	Standard		514.3	106.902	4	-10	-78	-14	-84	0.25	8%	10%
Tauro-beta-muricholic acid	TwMCA	Standard		514.3	106.9	4	-10	-73	-14	-84	0.25	14%	5%
Tauro-omega-muricholic acid	TbMCA	Standard		514.3	106.903	4	-10	-77	-14	-84			
Glycodeoxycholic acid	GDHCA	Standard		458.3	74	2.8	-10	-58	-14	-72	0.25	5%	17%
Taurohyocholic acid	THCA	Standard		514.3	106.901	4.42	-10	-77	-14	-72	1	11%	17%
Tauroursodeoxycholic acid	TUDCA	Standard		498.3	106.9	4.44	-10	-70	-14	-94	0.5	7%	30%
Taurohyodeoxycholic acid	THDCA	Standard		498.3	80.001	4.56	-10	-74	-14	-94	0.5	15%	10%
Taurocholic acid	TCA	Standard		514.3	124	4.82	-10	-71	-14	-72	1	30%	17%
Taurocholic acid-d5	TCA-d5	Internal standard	62.5	519.3	124	4.82	-10	-65	-14	-72			
Dehydrocholic acid	DHCA	Standard		401.3	331.2	3.21	-10	-34	-14	-52	0.25	2%	24%
Glycoursodeoxycholic acid	GUDCA	Standard		448.3	74.001	4.47	-10	-60	-14	-52	0.25	8%	3%
Glycoursodeoxycholic acid-d4	GUDCA-d4	Internal standard	62.5	452.3	74.002	4.47	-10	-83	-14	-78			
Omega-muricholic acid	wMCA	Standard		407.3	387.2	4.49	-10	-55	-14	-48	10	12%	34%
Glycohyocholic acid	GHCA	Standard		464.3	74.003	4.46	-10	-64	-14	-72	0.5	11%	19%
Glycohyodeoxycholic acid	GHDCA	Standard		448.3	74.004	4.6	-10	-60	-14	-72	0.025	5%	4%
Alpha-muricholic acid	aMCA	Standard		407.3	387.201	4.98	-10	-48	-14	-48	100		
Glycocholic acid	GCA	Standard		464.3	74.005	4.88	-10	-65	-14	-84	0.25	8%	3%
Glycocholic acid-d4	GCA-d4	Internal standard	62.5	468.3	74.006	4.88	-10	-88	-14	-84			
7-Oxodeoxycholic acid	7-OXO-DCA	Standard		405.3	123.1	4.47	-10	-55	-14	-76	0.25	10%	5%
Beta-muricholic acid	bMCA	Standard		407.3	371.2	5.44	-10	-48	-14	-62	10	33%	1%
Taurochenodeoxycholic acid	TCDCA	Standard		498.3	124	5.4	-10	-70	-14	-90	0.25	12%	6%
7-Oxohyocholic acid	7-oxo-HDCA	Standard		405.3	375.3	4.47	-10	-46	-14	-76	10	29%	14%
Hyocholic acid	HCA	Standard		407.3	389.2	4.98	-10	-47	-14	-50	10	14%	19%
Taurodeoxycholic acid	TDCA	Standard		498.3	124.001	5.59	-10	-67	-14	-90	0.25	14%	17%
Ursodeoxycholic acid-d4	UDCA-d4	Internal standard	62.5	395.3	395.3	4.96	-10	-30	-14	-90			
Ursodeoxycholic acid	UDCA	Standard		391.3	391.3	4.96	-10	-27	-14	-90	0.5	6%	6%
Cholic acid	CA	Standard		407.3	343.2	5.44	-10	-49	-14	-76	0.5	13%	7%
Cholic acid-d4	CA-d4	Internal standard	62.5	411.3	347.1	5.44	-10	-47	-14	-84			
Cholic acid-c13	CA-c13	Internal standard	62.5	408.3	343.3	5.44	-10	-49	-14	-76			
Hyodeoxycholic acid	HDCA	Standard		391.3	391.303	5.17	-10	-21	-14	-90	0.5	9%	4%
Glycochenodeoxycholic acid	GCDCA	Standard		448.3	74.008	5.49	-10	-63	-14	-68	0.5	19%	5%
Glycochenodeoxycholic acid-d4	GCDCA-d4	Internal standard	62.5	452.3	74.009	5.49	-10	-61	-14	-68			
Glycodeoxycholic acid	GDCA	Standard		448.301	74.01	5.69	-10	-55	-14	-68	0.25	4%	1%
12-Oxolithocholic acid	12-OXO-LCA	Standard		389.3	389.3	5.25	-10	-14	-14	-28	0.5	23%	44%
Taurolithocholic acid	TLCA	Standard		482.3	124	6.27	-10	-66	-14	-80	0.25	5%	7%
Chenodeoxycholic acid-d4	CDCA-d4	Internal standard	62.5	395.3	395.301	6.33	-10	-40	-14	-88			
Chenodeoxycholic acid	CDCA	Standard		391.3	391.301	6.33	-10	-7	-14	-88	0.5	23%	9%
Deoxycholic acid	DCA	Standard		391.3	391.302	6.53	-10	-23	-14	-88	10	1%	1%
Deoxycholic acid-d4	DCA-d4	Internal standard	62.5	395.3	395.302	6.53	-10	-45	-14	-88			
Glycolithocholic acid	GLCA	Standard		432.3	74.011	6.38	-10	-60	-14	-32	0.25	2%	3%
Glycolithocholic acid-d4	GLCA-d4	Internal standard	62.5	436.3	74.012	6.38	-10	-74	-14	-32			
Lithocholic acid	LCA	Standard		375.3	375.3	7.52	-10	-13	-14	-46	1		98%
Lithocholic acid-d4	LCA-d4	Internal standard	625	379.3	379.3	7.52	-10	-45	-14	-46			
Taurocholic acid-d4	TCA-d4	Internal standard	62.5	518.3	124.1	4.82	-10	-65	-14	-72			

Stool sampling, processing, and analysis

Stool samples were collected pre-dose in ethanol tubes by each volunteer at home and posted to the Oxford Centre for Microbiome Studies where they were stored at −80 °C. The samples were stored for two months before DNA extraction. Frozen samples were shipped to Transetyx (Cordova,TN), where DNA was extracted. Faecal microbiome composition was determined with shallow shotgun whole genome sequencing by Transnetyx (Cordova,TN). In brief, DNA underwent quality control and was then converted into sequencing libraries with unique dual indexed (UDI) adapters; libraries were sequenced by shotgun shallow metagenomic (depth: 2 million 2 x 150 bp read pairs) with with NovaSeq (Illumina, San Diego, CA); FASTQ files containing sequencing data were uploaded and analysed into the OneCodex analyses software, from which we obtained abundance and taxonomic Amplicon Sequence Variance (ASVs). ASVs data were converted into relative abundances and log-transformed. The Shannon index was used as a measure of within-individual diversity (alpha). The first three axes of principal component analysis of the beta diversity (Weighted and Unweighted UniFrac metrics) were extracted to represent between-individual differences in overall microbiome composition. Functional metagenomics analyses were conducted on the same samples using the Humann3 pipeline. Gene family data was annotated into Kegg metabolic pathways and converted into Copies Per Million (CPM) abundances. Functional analyses were used to estimate the CPM of two enzymes involved in the synthesis of secondary bile acids: Bile salt hydrolase (BSH, EC 3.5.1.24) and 7-alpha dehydroxylase (7αDH, EC 1.1.1.159). Functional analyses were completed by measuring secondary bile acids from the faecal samples at the TMC using a validated quantitative targeted method without measuring the PFAS compounds which are not present in high concentration in the faeces [2](see ref. for the full list), which were log-transformed before being entered in the analysis [3].

Microbiome data (ASVs, secondary bile acids, and CPM abundances) were used in linear regression analyses with CBD AUC as outcome. P values were adjusted for multiple testing using the false discovery rate method.

Supplementary Materials: Novel Lipid Formulation Increases Absorption of Oral Cannabidiol (CBD)

Supplementary Results

Treatment-emergent and treatment-related adverse events

Table S3. Treatment-emergent and treatment-related adverse events						
	Treatment-emergent adverse events			Treatment-related adverse events		
Adverse event	Lipid formulation (n=14)	Powder CBD formulation (n=14)	P value	Lipid formulation (n=14)	Powder CBD formulation (n=14)	P value
Participant with Any	10 (71.4%)	8 (57.1%)	0.48	8 (57.1%)	8 (57.1%)	1.00
Gastrointestinal disorders	5 (35.7%)	2 (14.3%)	0.18	5 (35.7%)	2 (14.3%)	0.18
Diarrhoea	2 (14.3%)	0 (0.0%)	0.16	2 (14.3%)	0 (0.0%)	0.16
Nausea	1 (7.1%)	1 (7.1%)	1.00	1 (7.1%)	1 (7.1%)	1.00
Vomiting	0 (0.0%)	1 (7.1%)	0.32	0 (0.0%)	1 (7.1%)	0.32
Flatulence	1 (7.1%)	1 (7.1%)	1.00	1 (7.1%)	1 (7.1%)	1.00
Abdominal distension	1 (7.1%)	0 (0.0%)	0.32	1 (7.1%)	0 (0.0%)	0.32
Abdominal pain	1 (7.1%)	0 (0.0%)	0.32	1 (7.1%)	0 (0.0%)	0.32
Neuropsychiatric disorders	8 (57.1%)	8 (57.1%)	1.00	6 (42.9%)	8 (57.1%)	0.53
Somnolence	5 (35.7%)	2 (14.3%)	0.257	3 (21.4%)	2 (14.3%)	0.65
Headache	2 (14.3%)	3 (21.4%)	0.56	2 (14.3)	3 (21.4%)	0.56
Dizziness	1 (7.1%)	1 (7.1%)	1.00	1 (7.1%)	1 (7.1%)	1.00
Relaxed	0 (0.0%)	1 (7.1%)	0.32	0 (0.0%)	1 (7.1%)	0.32
High	0 (0.0%)	1 (7.1%)	0.32	0 (0.0%)	1 (7.1%)	0.32
Anxiety	0 (0.0%)	0 (0.0%)	-	0 (0.0%)	0 (0.0%	-
Vasovagal	0 (0.0%)	0 (0.0%)	-	0 (0.0%)	0 (0.0%)	-

Gastrointestinal symptoms

Table S4. Gastrointestinal symptoms measured by the GSRS (n=14)						
	Lipid formulation (mean, SD)			Powder formulation (mean, SD)		
Symptom Domain	Pre-dose	24 hours	48 hours	Pre-dose	24 hours	48 hours
Reflux (2 items)	1.04 (0.13)	1.04 (0.13)	1.11 (0.29)	1.04 (0.13)	1.00 (0.0)	1.00 (0.0)
Abdominal pain (3 items)	1.24 (0.50)	1.38 (0.61)	1.14 (0.39)	1.10 (0.16)	1.21 (0.46)	1.02 (0.09)
Indigestion (4 items)	1.13 (0.27)	1.30 (0.53)	1.11 (0.21)	1.13 (0.19)	1.09 (0.21)	1.05 (0.11)
Diarrhoea (3 items)	1.00 (0.0)	1.43 (0.89)*	1.07 (0.27)	1.00 (0.0)	1.00 (0.0)*	1.00 (0.0)
Constipation (3 items)	1.07 (0.19)	1.10 (0.20)	1.00 (0.0)	1.05 (0.12)	1.00 (0.0)	1.02 (0.09)
Total score	1.09 (0.17)	1.25 (0.36)	1.09 (0.18)*	1.06 (0.09)	1.06 (0.11)	1.02 (0.04)*
*p<0.05, Wilcoxon signed-rank test						

REFERENCES

1. Dickens, A.M.; Borgan, F.; Laurikainen, H.; Lamichhane, S.; Marques, T.; Rönkkö, T.; Veronese, M.; Lindeman, T.; Hyötyläinen, T.; Howes, O.; et al. Links between Central CB1-Receptor Availability and Peripheral Endocannabinoids in Patients with First Episode Psychosis. *NPJ Schizophr* **2020**, *6*, 1–10, doi:10.1038/s41537-020-00110-7.
2. Salihović, S.; Dickens, A.M.; Schoultz, I.; Fart, F.; Sinisalu, L.; Lindeman, T.; Halfvarson, J.; Orešič, M.; Hyötyläinen, T. Simultaneous Determination of Perfluoroalkyl Substances and Bile Acids in Human Serum Using Ultra-High-Performance Liquid Chromatography–Tandem Mass Spectrometry. *Anal Bioanal Chem* **2020**, *412*, 2251–2259.
3. Lamichhane, S.; Sen, P.; Dickens, A.M.; Alves, M.A.; Härkönen, T.; Honkanen, J.; Vatanen, T.; Xavier, R.J.; Hyötyläinen, T.; Knip, M.; et al. Dysregulation of Secondary Bile Acid Metabolism Precedes Islet Autoimmunity and Type 1 Diabetes. *Cell Rep Med* **2022**, *3*, doi:10.1016/j.xcrm.2022.100762.
